# Correspondence regarding "Effect of active smoking on the human bronchial epithelium transcriptome"

**DOI:** 10.1186/1471-2164-10-82

**Published:** 2009-02-18

**Authors:** Scott D Zuyderduyn

**Affiliations:** 1 Victor Ling Laboratory, Department of Cancer Genetics and Developmental Biology, BC Cancer Research Centre, Vancouver, Canada; 2 Graduate Program, Department of Biochemistry and Molecular Biology, Faculty of Medicine, University of British Columbia, Vancouver, Canada

## Abstract

**Background:**

In the work of Chari *et al. *entitled "Effect of active smoking on the human bronchial epithelium transcriptome" the authors use SAGE to identify candidate gene expression changes in bronchial brushings from never, former, and current smokers. These gene expression changes are categorized into those that are reversible or irreversible upon smoking cessation. A subset of these identified genes is validated on an independent cohort using RT-PCR. The authors conclude that their results support the notion of gene expression changes in the lungs of smokers which persist even after an individual has quit.

**Results:**

This correspondence raises questions about the validity of the approach used by the authors to analyze their data. The majority of the reported results suffer deficiencies due to the methods used. The most fundamental of these are explained in detail: biases introduced during data processing, lack of correction for multiple testing, and an incorrect use of clustering for gene discovery. A randomly generated "null" dataset is used to show the consequences of these shortcomings.

**Conclusion:**

Most of Chari *et al.*'s findings are consistent with what would be expected by chance alone. Although there is clear evidence of reversible changes in gene expression, the majority of those identified appear to be false positives. However, contrary to the authors' claims, no irreversible changes were identified. There is a broad consensus that genetic change due to smoking persists once an individual has quit smoking; unfortunately, this study lacks sufficient scientific rigour to support or refute this hypothesis or identify any specific candidate genes. The pitfalls of large-scale analysis, as exemplified here, may not be unique to Chari *et al*.

## Background

I read with interest the recent work, published in *BMC Genomics*, entitled "Effect of active smoking on the human bronchial epithelium transcriptome" [1]. In this article, the authors present an analysis of gene expression profiles generated using serial analysis of gene expression (SAGE) [2]. The profiles were obtained from samples of the lung epithelium taken from individuals who have never smoked, had quit smoking, or are current smokers. The authors highlight the differences in gene expression between the three groups, with a particular focus on a substantial number of changes that appear to persist after an individual has stopped smoking. These results are intriguing, as they suggest that some etiology is permanently maintained after smoking cessation, that this manifests itself at the level of gene expression, and that such changes may contribute to an increased risk of developing lung cancer. The findings were widely reported in the mainstream media [3,4]. However, I have serious concerns about the statistical methods the authors employed and, consequently, the validity of the conclusions drawn.

## Main criticisms

My critique follows the order of results presented in the original paper. A summary of the issues addressed in this correspondence and their consequences are given in Table 1. In many cases, the authors draw inferences from their analysis that can be explained by chance alone. To demonstrate this, I created a null (or random) dataset that could be analyzed with the approaches used in Chari *et al. *This "null" dataset was generated by combining the SAGE data from all 24 of their analyzed samples and then randomly re-distributing this "meta-transcriptome" into libraries with sizes equal to those in the original dataset. The organization of these virtual libraries into never, former, and current smoker sub-categories is identical to the original dataset. Thus, the reference dataset retains the effects introduced by having unequal library sizes, unequal group sizes, and a range of total signal for each tag. The most important feature of the null dataset is that *any correlations of tag expression values with smoking status have occurred by chance alone*.

**Table 1:** Summary of criticisms.

**Flaw**	**Consequence**
poor definition of "preferential" expression	introduces unchecked bias from different group sizes

incorrect use of Venn diagram	confounds overall sense of group-specific differences

use of raw tag counts to determine "preferential" expression	introduces unchecked bias from different library sizes

data filtered using criteria that includes variable to be tested	pre-selects for data more likely to be found significant, confounding estimated of false discovery rate (FDR)

significance threshold set to *p *≤ 0.05 without adjusting for multiple testing	false discovery rate (FDR) could be very high

"significant" results undergo *post hoc *fold-change filter	low tag counts more likely to pass the filter, yet these more likely to represent random variation

other possible null hypotheses not tested	not possible to check for consistency with known biology

null hypotheses formed with 2 of the 3 sample types	loss of power

data selected for differential expression is clustered	formation of distinct clusters is meaningless

genes tested for consistency with third sample group restricted to genes pre-selected as different between original two groups	flaws in implementation of first hypothesis test become propagated and amplified in second hypothesis test

no RT-PCR of irreversible genes	no validation of irreversible gene expression hypothesis

evidence for GSK3B as an irreversible gene is weak or supports reversible hypothesis	selection of GSK3B for further experimentation is not indicated

tags per million (TPM) used in statistical testing rather than for reporting purposes only	artificially inflates non-zero counts

some SAGE tags incorrectly mapped	a) follow-up RT-PCR is not validation, b) evidence for involvement of COX2 pathway is weaker than implied

A short description of each of the criticisms addressed in this correspondence, and their consequences for Chari *et al*.

### The relationship between the transcriptomes of never, former, and current smokers

Chari *et al. *show the gross relationship between sample types in terms of the number of tags preferentially expressed in different subsets of the three smoking status groups (never, former, and current smokers). This is often seen in published SAGE studies, and it serves to orient the reader to the data and provide a preliminary sense of group-specific differences. A Venn diagram, like that found in Chari *et al.*, is a popular way of visualizing the results. However, there are two concerns with the authors' particular application of this method.

First, the criterion for "preferential" expression is unconventional. Specifically, they state "*the criteria chosen for preferential expression was a threshold of a raw tag count of ≥ 2 across all samples in a particular set, but not existing in the other sets*". This would seem to imply that a tag is preferentially expressed if it appears at the given threshold in all of the libraries of one group, and is below the threshold in all of the libraries of the other two groups. However, an inspection of the authors' supplementary material reveals that "not existing in other sets" can mean that a raw tag count of 0 or 1 appears in *any *library in both of the competing groups. For example, tag CGGTTTGCAT is considered preferentially expressed in former smokers and has the following counts: current = (1, 16, 12, 5, 17, 14, 16, 8), former = (19, 11, 8, 11, 2, 8, 5, 7, 6, 8, 3, 11), and never = (15, 0, 11, 11). However, the average expression in each group is: current = 100.7 TPM, former = 59.9 TPM, and never = 67.6 TPM. This problem is quite pronounced for a significant fraction of tags preferentially expressed in each of the never, former, and current smoker groups: 57/227 (25%), 33/102 (32%), and 635/2013 (32%), respectively, actually have a higher mean expression in one or both of the competing groups!

**Figure 1:**
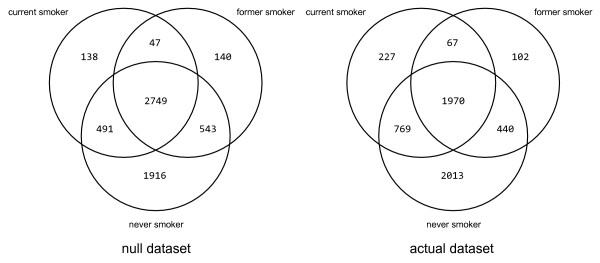
**"Venn" diagram using Chari *et al. *methodology on the null and the actual dataset**. The left hand Venn diagram shows values obtained using a null (randomized) dataset, and the right hand Venn diagrams shows values obtained using the actual dataset. Note these do not represent properly formed Venn diagrams (see text for details).

Second, even if the above approach was valid, there are severe biases introduced as a result of unequal library and group sizes. The former bias is exacerbated because the authors utilize raw tag counts in their comparisons. This means that a tag count of 2 from a library of 50,000 tags is equivalent to a tag count of 2 from a library of 200,000 tags. In the latter, the exclusion criterion (a 0 or 1 in at least one library) is more easily met for groups that have more libraries. I applied the same procedure to the "null" dataset, and the resulting diagram shows similar values to those obtained from the real data (Figure 1). In particular, the small number of never smoker libraries results in a large estimate of the "preferentially" expressed never smoker tags.

For these reasons, the results reported by the authors are of little use in determining a relationship between never, current and former smoker transcriptomes. An alternative would be to use much higher expression thresholds that apply equally to all libraries within a group to reduce the effects of sampling variation and differences in library and group sizes (e.g. ≥50 TPM versus ≤10 TPM). Principal component analysis or hierarchical clustering of the data are also good alternatives to visualize the overall relationship between profiles.

### Multiple testing and the determination of differentially expressed tags

In a review of microarray studies used to explore cancer outcomes, Dupuy and Simon identified the failure to adequately control for false positives as a result of multiple testing as the "most common and serious" flaw they observed in procedures for gene selection [5]. The problem can be grasped intuitively when considering a hypothesis test of 10,000 genes (or, in this case, tags). If one considers a *p*-value ≤ 0.05 as being significant, then we expect that 500 genes will be identified by chance alone. If 1500 genes are subsequently identified from the actual data, we can estimate that 500/1500 = 33% of these will be false positives. That this problem remains an issue in published studies is truly unfortunate, since a range of methods are available to overcome this problem. For instance, a simple Bonferroni correction or the Benjamini-Hochberg method are widely accepted and commonly used [6,7]. These methods can provide corrected *p*-values or estimates of the false discovery rate (FDR) for a given threshold *p*-value. Moreover, powerful resampling approaches (like that used to estimate the FDR in this correspondence) are precise and increasingly common with the rise in available computing power. Regardless of the method used, the objective is to determine a statistical cutoff that results in a reasonable number of false positives. An acceptable adjusted *p*-value or FDR is somewhat arbitrary, but for the latter metric a value lower than 10–20% is commonly cited [5].

In Chari *et al.*, the authors' failure to account for multiple testing or to provide some estimate of the FDR drastically diminishes the value of their findings. The authors use a Mann-Whitney U statistic (a non-parametric test) on each tag to calculate the *p*-value for the null hypothesis that never smokers are the same as current smokers. The authors restrict their testing to tags with a "mean tag count of ≥20 tags per million (TPM) in at least one of never, former or current smoker SAGE libraries". This introduces a bias by pre-selecting tags for testing based on criteria that includes the variable being tested (in this case, smoking status). This bias could be reasonably addressed by filtering using a criterion of a mean expression of 20 TPM across *all *libraries.

In any event, this filter produces a reduced test set of 8,148 tags to which the statistic is applied. The authors select those tags with *p *≤ 0.05 and then apply a fold-change criterion of ≥2. Before more fully exploring the central issue of multiple testing, it should be briefly mentioned that the fold-change criteria is somewhat dubious. It is easier for tags with low counts to meet this threshold than for those with higher counts. For example, assuming libraries consisting of 100,000 tags, a count of 1 versus a count of 3 represents a 3-fold change (*p *= 0.62, χ^2^-test), while the more significant difference of a count of 50 versus a count of 75 represents only a 1.5-fold change (*p *= 0.032, χ^2^-test). This is a concern when using a non-parametric test like the Mann-Whitney U with SAGE, since the relative rank of expression rather than the actual difference is used to provide statistical inference.

To demonstrate the larger problem of multiple testing, I compared the number of tags that meet a given threshold *p*-value in the actual dataset against the average number obtained from the null dataset in order to estimate the FDR. One must also use the number of results found prior to the use of a *post hoc *filter like fold-change, and I do so here. It's not clear from Chari *et al*. whether all 8,148 tags or a smaller number representing a minimum of 20 TPM in only the never or current smoker groups were used. In my estimates of the FDR, I use the more generous assumption of the latter (7,764 tags). Of these, 885 tags have *p *≤ 0.05 and 609 of these pass the fold-change filter. When this identical analysis is applied to the null dataset, 7,406 tags are expressed at a mean of 20 TPM in either the never or current smoker groups. Of these, 418 tags have *p *≤ 0.05 and 195 of these pass the fold-change filter. Therefore, we can estimate the FDR as 418/885 = 47.2%. This means that, at a minimum, nearly half of the authors' findings are false positives. The true FDR will be higher due to the initial biased filter of group-specific expression of ≥20 TPM and still higher in the reduced set of 609 tags because of the bias introduced by the fold-change filter.

**Figure 2:**
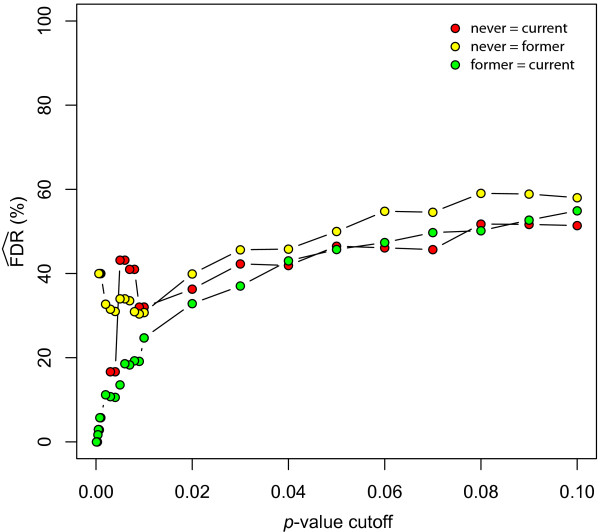
**Estimated false discovery rate at different threshold p-values for comparisons of two groups**. The x-axis represents different *p*-value cutoffs (0.00–0.10) to determine differential expression, and the y-axis represents the estimated false discovery rate (FDR) expected for each cutoff. The color key to differentiate the three possible null hypotheses comprising two of the three groups is shown in the legend.

**Figure 3:**
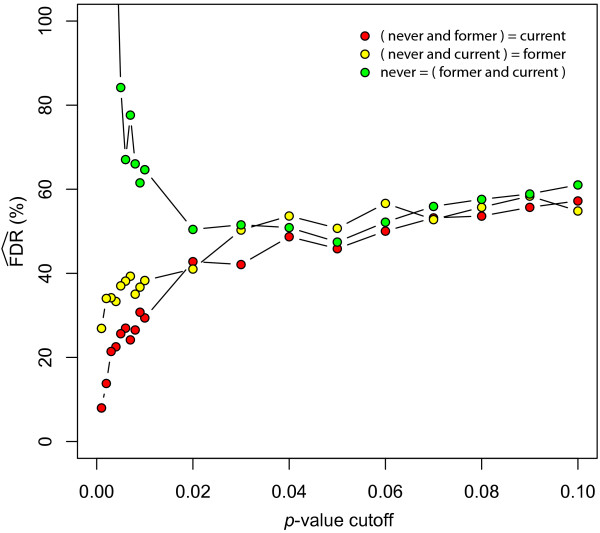
**Estimated false discovery rate at different threshold p-values for comparisons of three groups**. The x-axis represents different *p*-value cutoffs (0.00–0.10) to determine differential expression, and the y-axis represents the estimated false discovery rate (FDR) expected for each cutoff. The color key to differentiate the three possible null hypotheses involving pair-wise combinations of all of the three groups is shown in the legend.

It is unfortunate that the authors only reported the findings of a test of one null hypothesis (N = C). The other null hypotheses of (N = F) and (F = C) were not discussed. When performing these comparisons using the real and null dataset, a disturbing trend emerges (Table 2). Specifically, the estimated FDR is fairly similar amongst all three of the possible comparisons at *p *≤ 0.05 (47.2%, 50.2%, and 46.5%). This would suggest that there are a similar number of tags with differential expression specific to each of the three groups. This presents a problem, since the authors' hypothesis of "reversible" and "irreversible" gene expression change would lead one to expect that never and current smokers would define the limits of expression, with former smokers falling somewhere within this continuum. In order to explore this further, I plotted the estimated FDR over a range of cutoff *p*-values to see if a clearer pattern would emerge with more stringent criteria (Figure 2). It can be clearly seen that as one approaches a more rigorous *p*-value cutoff, the FDR falls below 20% for both the N = C and F = C null hypotheses, but remains at around 40% for the N = F null hypothesis. One must keep in mind that the number of samples in each group will affect the limits of confidence that can be obtained in their corresponding hypothesis test, but it seems clear that the current smoker group is similarly different from either never or former smokers. This can be explored more directly by formulating null hypotheses based on *all *of the groups. Indeed, this seems to be the most natural way of identifying "reversible" and "irreversible" gene expression differences. Three null hypotheses are possible: (N, F = C), (N = F, C), (N, C = F). The first two would correspond to "reversible" and "irreversible" changes, respectively, while the third does not correspond with the expected biology of smoke-exposure and could act as an internal control (i.e. this comparison would be expected to produce comparatively fewer results than the other two). When I performed this analysis using the authors' methods, the concerns alluded to in the two-group comparisons become all too clear (Table 2). The only null hypothesis where a correction for multiple testing can yield a FDR ≤20% is (N, F = C), which corresponds to "reversible" changes. (N = F, C), which corresponds to "irreversible" changes, has an abysmal FDR once a cutoff of about *p *≤ 0.01 is established (Figure 3). Even the hypothesis test for genes specific to former smokers, (N, C = F), performs better.

**Table 2:** Estimated false discovery rate (FDR) of null hypotheses using different combinations of the three groups

	null dataset			actual dataset			
null hypothesis	≥20 TPM	p ≤ 0.05	fold-change ≥ 2	≥20 TPM	p ≤ 0.05	fold-change ≥ 2	FDR

N = C	7406	418	195	7764	885	609	47.2
N = F	7323	384	157	7547	765	447	50.2
F = C	7102	416	92	7318	895	433	46.5
(N, F) = C	7726	411	82	8148	959	460	42.8
N = (F, C)	7726	382	67	8148	836	475	45.7
(N, C) = F	7726	388	157	8148	818	314	47.4

The estimated false discovery rate (FDR) of different null hypotheses when grouping the 24 SAGE libraries of Chari *et al. *into never, former, and current smokers. A *p*-value cutoff of 0.05 was used, as in the original paper.

There do appear to be changes that correlate with the *individual *groups, since the estimated FDR is well below 100%. However, the differences are clearly difficult to distinguish from random variation in the dataset and most certainly do not support the irreversible/reversible dichotomy imposed by the authors. Most likely, there are other explanatory variables that would better explain the structure of the data. If such covariates were even weakly correlated with smoking status, they may well produce the results shown in this correspondence. In fact, several enticing variables are listed in the authors' own sample descriptions: age, pack-years, lung function, and years since quitting.

### Supervised clustering of never, former, and current smoker profiles

Chari *et al. *use a technique they refer to as "supervised clustering". Dupuy and Simon describe this approach as the "most common and serious flaw" when performing class discovery [5]. Specifically, one cannot state that clusters corresponding with a variable (i.e. smoking status) are meaningful if the input genes were selected for a correlation with the same variable. In Chari *et al.*, the authors cluster the 609 tags selected based on their differential expression between never and current smokers. Since the tags were selected based on this very property, it is not surprising that distinct clusters emerge; however, these clusters are not meaningful.

**Figure 4:**
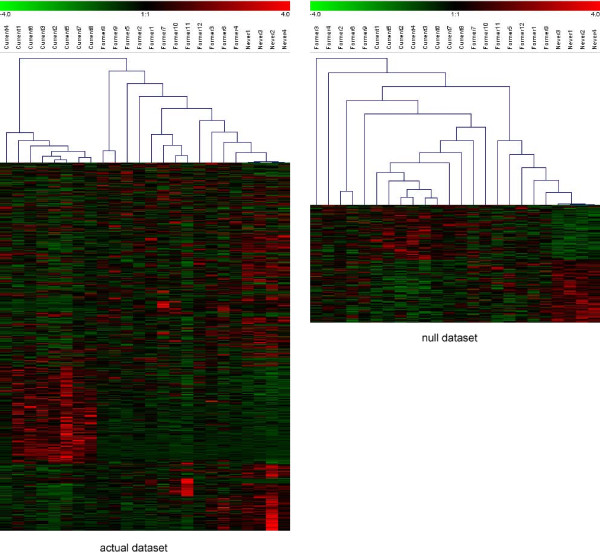
**Hierarchical clustering using Chari *et al. *methodology on the null and the actual dataset**. Counts for SAGE tags that met a threshold *p*-value cutoff of 0.05 for the null hypothesis of never = current smokers. The counts were row normalized and underwent single-link hierarchical clustering using a Pearson correlation as a distance metric. The left-hand tree represents the actual dataset and the right-hand tree represents the null dataset.

The authors go a step further by adding the expression data for the former smokers and then claiming that the clustering of *all three *is meaningful (authors' Figure 2A). If one was to generate random data for three equal-sized groups, select genes that are the most differentially expressed between two of these groups, and then proceed to cluster the data for all three, one would expect a similar pattern to that observed by the authors to emerge. However, in this case, the dataset has unequal library and group sizes and so the null clustering pattern will be affected. I was unable to reproduce the exact hierarchical clustering pattern in Chari *et al. *for their 609 differentially expressed tags, despite using the Genesis software to perform a single-link clustering with Euclidean distance (as described in the paper) [8]. After exhaustive trials using different program options, it was found that row normalization of the data followed by single-link clustering with a Pearson correlation distance metric yielded the most similar results. When I clustered the 195 tags from a representative resampling in the null dataset that were differentially expressed between never and current smokers, two distinct clusters emerged (Figure 4). The never smokers clustered tightly together, whereas the current and former smokers formed a distinct, but more diffuse, cluster. The clustering pattern observed for the authors' real data actually argues that former smokers are more similar to never smokers than expected by chance. Specifically, in contrast to the null clustering pattern, the former smoker libraries are distinct from current smokers and are more similar to the never smoker cluster.

For these reasons, this section of the authors' results is almost meaningless. The little inference that can be made actually indicates a situation at odds with the authors' stated conclusions. Hierarchical clustering and principal component analysis are powerful techniques in class discovery and, as alluded to previously, would have been better applied at the beginning of the authors' paper in an unsupervised manner on an unbiased set of data that does not consider smoking status.

### Additional criticisms

Although the key concerns have been described above, a number of additional issues with the authors' selection of irreversible and reversible genes, as well as the RT-PCR validation efforts, were removed for brevity. These have been included as a supplementary document (see Additional File 1).

## Conclusion

Large-scale data analysis is not trivial and flaws are often found in the published literature. It is unfortunate that this perceived complexity and unreliability of large data set analyses have caused many to be wary of gene expression studies when they hold so much potential to provide new insights. Many of the pitfalls of a large-scale analysis can be avoided by keeping a few basic tenets in mind. The data as presented supports the idea that changes to gene expression occur as a result of active smoking, but provides no compelling evidence that these changes persist once an individual has quit. Given that this study was widely publicized, these critical shortcomings become even more important to communicate. This is not to say that such permanent changes do not exist, or that such changes are not identifiable from the dataset the authors use. However, my perspective is that Chari *et al. *requires major revisions to support their stated conclusions or to consider the work as a basis for decision making in the design of future research.

## Authors' contributions

SDZ wrote the correspondence and performed the supporting analyses.

## Response from original authors

Raj Chari, Raymond T. Ng

Email: rchari@bccrc.ca

The analysis of high throughput gene expression data has been a challenging endeavor in the field of genomics. Numerous techniques and approaches have been developed and utilized, with each method having its own advantages and disadvantages. In an extensive analysis of the statistical approaches used in our original study [1], Zuyderduyn claimed to have derived different conclusions. Specifically, the existence of genes irreversibly expressed upon smoking cessation.

With respect to the statistical analysis, two of the main criticisms raised were the lack of using a multiple hypothesis testing correction and the use of the Venn diagram in one of our display items. The purpose of using a multiple hypothesis testing correction is to reduce potential false positives and Zuyderduyn demonstrates by randomly permuting the dataset, there would be a high degree of false positives at the statistical thresholds we employed. Given that there have only been a handful of studies to date examining gene expression changes associated with smoking in the bronchial epithelium, it is difficult to ascertain whether these false positives are indeed false positives. Those changes which corroborate between studies tend to agree with those which we have place the most confidence. In a study by Beane *et al *published after ours [9], where the issue of reversibility and irreversibility is investigated at a greater detail, we see a large number of genes which we identified in our study which were also identified in this study. In addition, it is important to emphasize that the genes which did not corroborate between both studies are not necessarily false positives, as subtler changes identified using one methodology may not always be readily detected by another. In terms of the Venn diagram, it was a simple illustration of genes expressed at different levels among different groups. Similar graphics were used by Shah et al to illustrate expression changes in current, former and never smokers in a previously published study [10]. This is a succinct way to summarize data.

There was also an issue of calling *GSK3B *as an irreversible gene. In a study by Spira *et al *[11], a gene with irreversible expression was defined as a gene whose expression was different between current and never smokers, but also different between former and never smokers. In our study, we illustrate the relationship between genes such as *CABYR*, *GSK3B*, *COX2*, *TFF3*, and *MUC5AC *and we show that the expression of three of the genes appeared to be significantly different between current and never smokers. Though *COX2 *expression was not evident, we observed an interesting trend of *GSK3B *expression. Subsequently, using a more precise experimental method, namely quantitative PCR, we assessed its expression in a second cohort with a larger panel of never smokers. We found that there was a statistically significant change in *GSK3B *expression between current and never smokers as well as former and never smokers. Hence, by the definition used above, this would qualify this gene as irreversible. It is important to reiterate that the key genes discussed in the paper such as *CABYR*, *TFF3*, *MUC5AC*, and *GSK3B *were not only assessed using SAGE, but were also assessed using quantitative PCR in a completely different cohort of samples.

In conclusion, though criticisms raised by Zuyderduyn are constructive and may serve as a cautionary note for gene expression data analysis in general, we firmly believe in the conclusions we have reached which were further supported by experimental evidence subsequently reported by other groups. Moreover, as with all large scale gene expression studies, it is useful to remember that genes identified at the first level of statistical analysis are in fact "best guesses". Further experimental verification is typically necessary to elucidate the relevance of the candidate genes to the disease state studied.

## Supplementary Material

Additional file 1 **Supplementary criticism**. A document describing some additional criticisms removed from the correspondence for brevity. Click here for file
