# Supplementary material for: Correspondence regarding "Effect of active smoking on the human bronchial epithelium transcriptome"
Source: BMC Genomics. 2009 Feb 18;10:82. doi: 10.1186/1471-2164-10-82 (PMC2656532; doi:10.1186/1471-2164-10-82)
Supplement: Additional file 1 — Supplementary criticism. A document describing some additional criticisms removed from the correspondence for brevity. [file 1471-2164-10-82-S1.pdf]

Supplementary Material

# Correspondence regarding “Effect of active smoking on the human bronchial epithelium transcriptome”

Scott D Zuyderduyn (scottz@bccrc.ca)

Selection of reversible and irreversible genes

Chari *et al.* restrict their search to the set of 609 tags differentially expressed between never and current smokers. Reversible genes are then considered to be those that show a "significant expression difference between current and former smokers" and, correspondingly, irreversible genes are those that show a significant different between never and former smokers. As stated previously, a more natural approach would seem to be to test the null hypotheses (N,F=C) and (N=F,C), respectively.

The authors make the questionable assumption that if a former smoker expressed tag is different from current smokers, it must be similar to never smokers, and is therefore "reversible" (and the same logic is used for "irreversible" tags). However, this doesn't account for the possibility that former smoker expression could be different from both. In fact, 13 of the tags appear to be both "irreversible" and "reversible". One cannot necessarily argue that these tags fall somewhere between never and current smokers and are therefore "partially reversible" since the authors do not report whether the former smoker expression values are either significantly higher or significantly lower than both of the other two groups.

The authors report finding 161 "reversible" tags and 152 "irreversible" tags. However, when I applied the equivalent analysis to the representative of the null dataset, a familiar theme emerges. Again, the proportion of "reversible" changes is somewhat greater than expected by chance, but the proportion of "irreversible" changes is not (Supplementary Table 1). Again, this re-enforces the notion that there are changes that result from active smoking, but the approach employed by the authors is not sufficient to make any argument for the existence of "irreversible" changes.

Validation of reversible and irreversible genes

It is quite striking how the validation efforts described by the authors support the criticisms I've outlined. There seemed to be little difficulty in validating "reversible" gene expression changes, and 3 such examples (CABYR, ENTPD8, TFF3) are shown. However, as expected, the authors could not validate any of the "irreversible" genes that were found. GSK3B, the one example given, was not a gene identified in their list of 152 "irreversible" genes. So, in fact, the RT-PCR results for GSK3B do not represent a validation, but a *de novo* hypothesis that still remains to be validated. The authors supply a box plot of the SAGE expression for GSK3B based on the counts of the tag CAATAAAGGT. The reported trend suggesting irreversible expression is based on average tag counts of 0.8, 1.6, and 3.3 (normalized to a library size of 100,000) in the current, former, and never smoker groups, respectively. The higher value for the current smokers is due to a single library. Regardless, the values are too low to distinguish from sampling variation. Neither SAGE Genie nor TagMapper, the two resources the authors report using for tag to gene mapping, report GSK3B as the gene corresponding to this tag (Boon, 2002; Bala, 2005). Full-length GSK3B has been sequenced and the canonical tag is CTCGGATTCA, which has a count of 1 in two of the 24 study libraries. Therefore, irrespective of the tag used, the data does not support the notion of GSK3B as an "irreversible" gene. A short

investigation of the SAGE tag CAATAAAGGT suggests that it is an artifact of the tag for the gene RPS8 (TAATAAAGGT), which is very highly expressed.

Moreover, the argument for pursuing GSK3B as an irreversible gene based on a role in the COX2 pathway is weak. The values provided in the authors' Figure 5 are expressed in tags per million, and many of the values cited represent extremely low expression values (e.g. 22 TPM is 2.2 tags in a typically sized library of 100,000 tags). An examination of the raw data for the tags corresponding to highlighted members of this pathway in fact indicates that either: a) no inference can be made, or b) the values support the "reversible" hypothesis. In addition to an incorrect tag mapping for GSK3B, the tag for EGFR also appears dubious (according to SAGE Genie, AGTACCTTAT maps to Hs.605083 which is annotated as "placenta mRNA, clone PL45, partial sequence").

Supplementary Table 1 – Evaluation of procedure to identify reversible and irreversible changes to gene expression as a result of smoke exposure

The procedure to identify reversible and irreversible changes used in Chari *et al.* is performed on the actual data and on a null (randomized) dataset. Of particular note is the similarity in the percentage of irreversible changes identified (27.7% versus 25.0% in the null and actual dataset, respectively).

**null hypothesis**	**null dataset**			**actual dataset**		
	tags tested	$p \leq 0.05$	fold-change≥2	tags tested	$p \leq 0.05$	fold-change≥2
F=C (“reversible”)	195	42 (21.5%)	19 (9.7%)	609	221 (36.3%)	161 (26.4%)
N=F (“irreversible”)	195	69 (35.4%)	54 (27.7%)	609	205 (33.7%)	152 (25.0%)

References

Boon K, Osorio EC, Greenhut SF, Schaefer CF, Shoemaker J, Polyak K, Morin PJ, Buetow KH, Strausberg RL, De Souza SJ, Riggins GJ. (2002) An anatomy of normal and malignant gene expression. *Proc Natl Acad Sci USA*, **99**:11287-11292.

Bala P, Georgantas RW, Sudhir D, Suresh M, Shanker K, Vrushabendra BM, Civin CI, Pandey A. (2005) TAGmapper: a web-based tool for mapping SAGE tags. *Gene*, **364**:123-129.
